# Mechanisms and Cardiorenal Complications of Chronic Anemia in People with HIV

**DOI:** 10.3390/v16040542

**Published:** 2024-03-30

**Authors:** Kingsley Kamvuma, Benson M. Hamooya, Sody Munsaka, Sepiso K. Masenga, Annet Kirabo

**Affiliations:** 1 HAND Research Group, School of Medicine and Health Sciences, Mulungushi University, Livingstone Campus, Livingstone 10101, Zambia; kamvumak@yahoo.com (K.K.); benmalambo@gmail.com (B.M.H.); 2 Department of Biomedical Sciences, School of Health Sciences, University of Zambia, Lusaka P.O Box 50110, Zambia; s.munsaka@unza.zm; 3 Vanderbilt Institute for Global Health, Vanderbilt University Medical Center, Nashville, TN 37232, USA; 4 Department of Medicine, Vanderbilt University Medical Center, Nashville, TN 37232, USA; 5 Vanderbilt Center for Immunobiology, Vanderbilt University Medical Center, Nashville, TN 37232, USA; 6 Vanderbilt Institute for Infection, Immunology and Inflammation, Vanderbilt University Medical Center, Nashville, TN 37232, USA

**Keywords:** anemia, chronic anemia, HIV, cardiovascular disease, kidney disease, people living with HIV, immune activation, inflammation

## Abstract

Chronic anemia is more prevalent in people living with HIV (PLWH) compared to the general population. The mechanisms that drive chronic anemia in HIV are multifaceted and include functional impairment of hematopoietic stem cells, dysregulation of erythropoietin production, and persistent immune activation. Chronic inflammation from HIV infection adversely affects erythropoiesis, erythrocyte lifespan, and erythropoietin response, leading to a heightened risk of co-infections such as tuberculosis, persistent severe anemia, and increased mortality. Additionally, chronic anemia exacerbates the progression of HIV-associated nephrotoxicity and contributes to cardiovascular risk through immune activation and inflammation. This review highlights the cardinal role of chronic inflammation as a link connecting persistent anemia and cardiovascular complications in PLWH, emphasizing the need for a universal understanding of these interconnected pathways for targeted interventions.

## 1. Introduction

An estimated 38.4 million people worldwide were living with human immunodeficiency virus (HIV) in 2021, and 1.5 million people were newly infected in the same year [1]. Hematological derangements are one of the most common complications among PLWH and have an impact on the quality of their lives [2]. The most common hematological complication of HIV is anemia, which has been associated with HIV disease progression and increased mortality [3,4,5]. According to the World Health Organization (WHO), anemia is a condition in which the number of red blood cells or the hemoglobin concentration within them is lower than normal, i.e., <12 g/dL in females and 13 g/dL in males [6]. The pathogenesis of anemia in HIV is multifactorial. PLWH are at higher risk of chronic anemia than the general population due to concomitant micronutrient deficiencies (especially iron), opportunistic infections, chronic immune activation and inflammation, antiretroviral therapy (ART), and HIV infection of hematopoietic stem cells (HSCs) [7].

Recent data have shed light on the complex mechanisms through which immune processes affect red blood cell production. Sub-optimal chronic inflammation is a hallmark of HIV infection that persists even in the presence of ART [8,9]. The virus itself triggers a cascade of immune responses, leading to a continuous state of inflammation [10]. This persistent immune activation is a result of the virus’s ability to evade immune surveillance. This may be in part due to the formation of viral reservoirs and the presence of viral antigens [11]. Consequently, immune cells are constantly activated, leading to the release of pro-inflammatory cytokines, particularly interleukin-6 (IL-6) and tumor necrosis factor-alpha (TNF-α) [12].

Chronic immune activation and inflammation are implicated in dysregulated iron metabolism and persistent anemia [13]. Anemia that develops in this context is formally known as anemia of chronic disease or anemia of inflammation. This anemia is associated with elevated concentrations of pro-inflammatory mediators, which can upregulate hepcidin production [14,15]. Hepcidin is a hormone produced by the liver that regulates iron homeostasis [16]. Hepcidin binds to ferroportin at numerous sites, including the gastrointestinal tract and reticuloendothelial system and limits the degree to which ferroportin is available to transport iron across the plasma membrane [17,18,19]. HIV can impair the survival/proliferative capacity of hematopoietic progenitor cells (HPC) [20,21,22]. Progressive depletion of HPCs or suppression of their function could result in hematologic abnormalities, such as chronic anemia, thrombocytopenia, and neutropenia [23]. HIV-1 strains also have an impact on the dynamics of CD34+, CD7+, and CD38+ hematopoietic progenitor cell pool, resulting in impaired T-cell production potential that further exacerbates HIV-induced anemia and disease pathogenesis [24].

The relationship between chronic kidney disease (CKD), chronic anemia, and cardiovascular risk in PLWH presents a multifaceted challenge in modern healthcare. As effective antiretroviral therapy (ART) prolongs the lives of PLWH, the evolving landscape of their health necessitates a comprehensive understanding of the sophisticated relationship between CKD, chronic anemia, and cardiovascular complications. This review elucidates the complicated immune mechanisms that contribute to chronic anemia in HIV patients, thereby providing insights that may appraise improved clinical management approaches. The interplay between chronic anemia, CKD, and cardiovascular risk in PLWH has been elaborated, addressing the evolving healthcare needs of this population.

## 2. Burden of Anemia in People with HIV

### 2.1. Prevalence of Anemia

Substantial variations in the prevalence of anemia in different countries in PLWH have been reported, ranging from 1.3% to 95% in different geographic settings [3,7,25,26]. Globally pooled prevalence for anemia is approximately 39.7% (95% CI: 31.4–48.0%) among children under the age of 15, 46.6% (95% CI: 41.9–51.4%) in adults (including both men and non-pregnant women) aged 15 years and older, and 48.6% (95% CI: 41.6–55.6%) in pregnant women [3]. The prevalence of anemia among adults living with HIV is higher in low and middle-income countries (LIMCs), especially in sub-Saharan Africa [27]. The prevalence of anemia is higher among adults with HIV in Southern Africa (58 to 70%) compared to those from East Africa (21 to 40%) [28,29,30,31,32]. For instance, a randomized clinical trial of ART efficacy in Africa, Asia, South America, the Caribbean and the USA showed that the prevalence of anemia differed significantly among these countries, and the prevalence was highest in Malawi, Haiti, South Africa, and Zimbabwe [33]. These differences across regions can be explained by the levels of poverty, malnutrition, and poor socioeconomic state of a given population.

### 2.2. Duration of ART and Anemia Risk

Approximately 95% of PLWH before the initiation of ART and up to 46% of PLWH taking ART develop anemia at some point during their disease [32]. Initiating ART early after HIV diagnosis, particularly before significant immune damage occurs, has been associated with an increase in hemoglobin levels [34]. Early ART can lead to improved immune function, reduced opportunistic infections, and better bone marrow function, which may contribute to the resolution of anemia [34,35]. PLWH may already have anemia at the time of HIV diagnosis due to the direct impact of the virus on bone marrow and red blood cell production [27,35]. Delayed HIV diagnosis and disease progression can contribute to more severe anemia before initiating ART [36,37].

With successful viral suppression and long-term adherence to ART, anemia can often improve [38]. As ART slows HIV replication and reduces the burden of opportunistic infections and inflammation, normal bone marrow function is restored, reducing the burden of anemia [7]. In some cases, patients may experience a transient worsening of anemia during the early weeks or months of ART initiation [32].

There are limited studies that evaluated the risk of chronic anemia with respect to the time and duration of ART. Harding et al. conducted a prospective clinical cohort study of adult PLWH receiving care at eight sites across the United States between 2010 and 2018 [7]. They found that among the 12,249 PLWH who were free of anemia at baseline, 265 developed chronic anemia (lasting for at least 6 months) during follow-up for an incidence of 0.46/100 people/year [7]. The incidence of anemia was 1.95/100 people/year, and the incidence of severe anemia was 0.68/100 people/year [7].

A study by Belay et al. revealed a high prevalence of anemia, reaching 54.9% among the participants [39]. The incidence rate of anemia was reported at 12.07 per 100 people/month, emphasizing the substantial risk associated with ART. Interestingly, the study suggests a temporal aspect, with 2.97% of women developing anemia in the first 6 months, escalating to 80.26% in the last 6 months of follow-up [39]. This temporal trend raises questions about the prolonged use of ART and its potential contribution to anemia development. However, there are more studies that support the idea that early ART reduces the incidence and prevalence of anemia. In a prospective study from China, the prevalence of anemia among 436,658 PLWH initiating ART was 29%, and annually, this prevalence reduced to 17.0%, 14.1%, 13.4%, 12.6%, and 12.7%, respectively [40]. This reduction in prevalence was reported in many studies. The persistence of anemia even after initiating ART is associated with being anemic at initiation, use of Zidovudine, malnutrition, low baseline weight at initiation, infection with tuberculosis, and female sex [27,32,40,41,42].

## 3. Underlying Mechanisms of Chronic Anemia in HIV

### 3.1. HIV Reactivation and Infection of Hematopoietic Stem Cells Mediated by the TNF-α-Dependent NF-κB Pathway

HSCs, residing in the bone marrow, are the architects of the blood cell repertoire, giving rise to various lineages, including red blood cells, white blood cells, and platelets [43]. Even when ART successfully suppresses plasma viral loads to undetectable levels, the recovery of HIV-1-related HSC proliferation and immune cell development is often incomplete [44]. This results in abnormalities of HSC development and differentiation, contributing to HIV-1 infection-induced immune pathogenesis in these patients [45]. The mechanisms for the dysfunctional hematopoiesis occurring during chronic HIV-1 infection are still unclear. However, HIV-1 infection may functionally impair hematopoietic progenitors through either viral products, induction of persistent inflammatory responses, or direct infection of HSCs [45].

HIV-1 infection may functionally impair HSC progenitors, potentially through the action of viral products that are released during infection [22,23]. These viral products, including but not limited to gp120 and transactivator of transcription (Tat), may play a role in disrupting the clonogenic capacity of bone marrow HSCs, contributing to abnormalities in the development and differentiation of HSCs [22,23,45], leading to chronic anemia and immune dysfunction. In addition, HIV-1 proteins such as Nef [46] and prolonged treatment with antiretroviral drugs could also compromise hematopoietic progenitors [47]. The exact mechanisms by which these viral products interfere with hematopoiesis are still under exploration, but their impact on HSC function can lead to disturbances in the delicate balance of blood cell formation.

**Figure 1:**
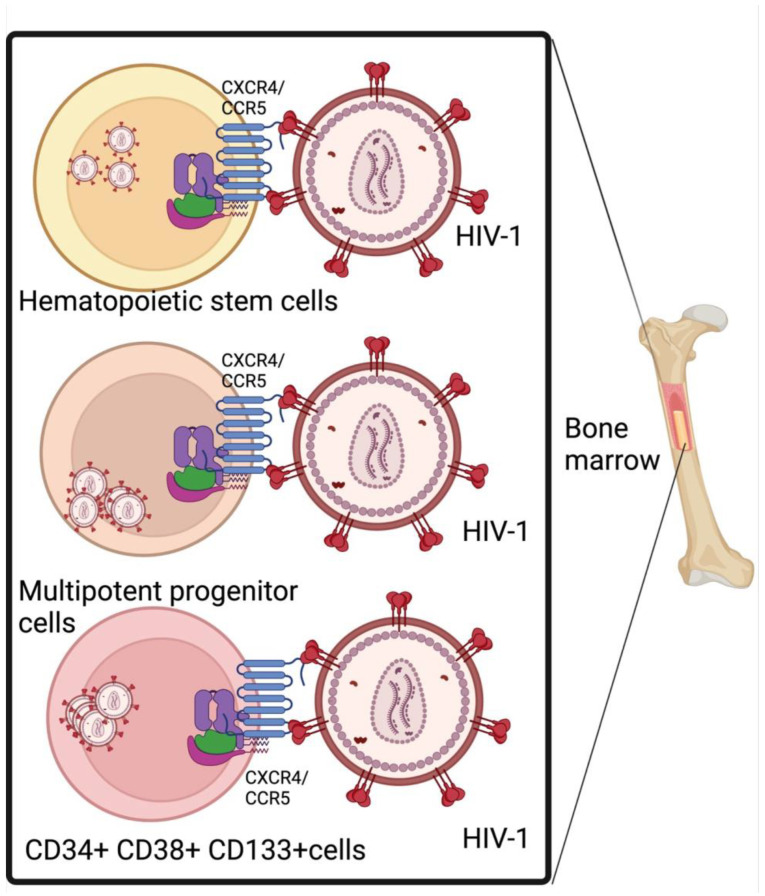
HIV-1 infection of hematopoietic stem and progenitor cells via specific receptors. Hematopoietic stem and progenitor cells are susceptible to HIV infection. HIV interacts with CCR5 and CXCR4 on stem cells in the bone marrow to disrupt hematopoiesis. CXCR4, C-X-C chemokine receptor type 4; CCR5, CXC motif chemokine receptor 5.

HIV-1 infection can functionally impair hematopoietic progenitors through direct infection of HSCs, leading to a disturbance in hematopoiesis [48]. HIV utilizes the CD4 receptor and chemokine co-receptors, such as C–C chemokine receptor type 5 (CCR5) or C-X-C chemokine receptor type 4 (CXCR4), also known as fusin or CD184, to enter HSCs [49]. Renelt et al. also demonstrated that HIV-1 can infect hematopoietic stem and progenitor cell (HSPC) subsets (CD34+CD38+ progenitors, MPPs, and HSCs) via host cell expression of CD4/CXCR4/CCR5 [49], as shown in Figure 1. However, the level of CXCR4/CCR5 expression in HSCs is low compared to immature and mature CD4+ cells [50]. Once inside, HIV-1 can establish a latent infection or integrate into the genome, potentially affecting the normal functioning of HSCs. Hematopoietic stem and progenitor cells and sites regulating hematopoiesis have been identified as one of the sites supporting HIV proviral integration contributing to clonal expansion and persistence of infected cells [51,52,53,54]. A study by McNamar et al. demonstrated that all subsets of HSCs (CD34+ CD38− CD45RA−), including immature HPCs (CD133+) and multipotent progenitors (MPPs), are latently infected by HIV-1 [55].

**Figure 2:**
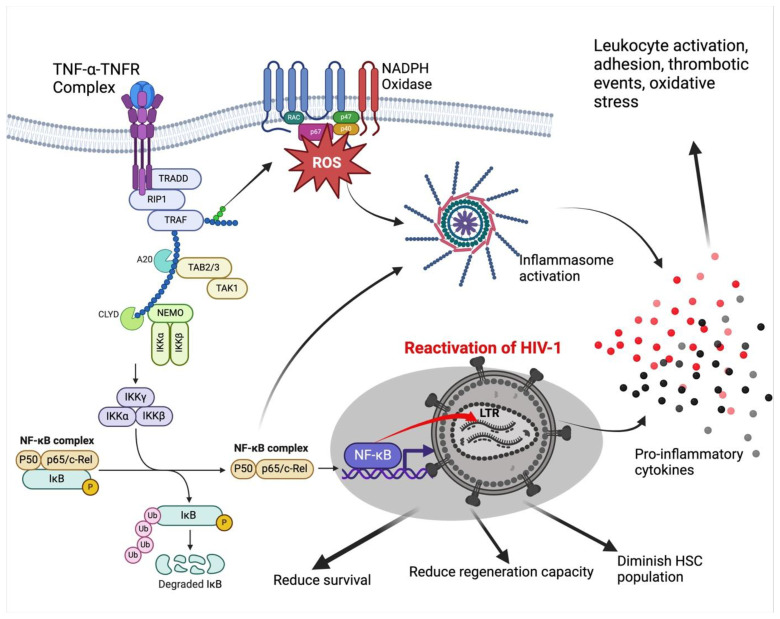
TNF-α-NF-κB-dependent mechanism of HIV reactivation. Cells infected with HIV-1 induce the production of TNF-α, which binds to TNF-α receptors, activating multiple signaling proteins with NF-kB recruitment and translocation into the nucleus to bind to LTR on HIV-1, reactivating it. This results in impaired HSC survival and regeneration and multiple inflammatory responses. TNF-α, tumor necrosis factor alpha; TNFR, tumor necrosis factor-alpha receptor; TRADD, TNF receptor-associated protein with a death domain; RIP1, receptor-interacting protein 1; TRAF, TNF receptor-associated factor; IκB inhibitor of nuclear factor-κB; IKK, inhibitor of nuclear factor-κB kinase; NEMO, NF-κB essential modulator; LTR, HIV-1 long terminal repeat; ROS, reactive oxygen species; NF-κB, Nuclear factor kappa B; TAK1, transforming growth factor-β-activated kinase 1; TAB, TAK-1-binding proteins.

HIV is reactivated by TNF-α through a nuclear factor kappa-light-chain-enhancer of activated B cells (NF-κB)-dependent mechanism [55]. In HIV-1 infection, TNF-α secretion by multiple infected cells, including activated macrophages and T cells, is induced by HIV viral proteins Tat and gp120 released extracellularly [56,57,58,59]. TNF-α binds to tumor necrosis factor receptors (TNFRs) on HSCs, initiating a dependent pathway by recruiting two adaptor proteins, a TNF receptor-associated protein with a death domain (TRADD) and serine–threonine kinase receptor-interacting protein 1 (RIP1) [60]. NF-κB is activated in this way through the classical pathway [61]. TRADD then recruits and activates the TNF receptor-associated factor (TRAF) and the β subunit of the inhibitor of nuclear factor-κB (IκB) kinase (IKK) [62]. IKK contains kinases such as IKK1 and IKK2 and NF-κB essential modulator (NEMO) [63,64]. The kinases phosphorylate and cause ubiquitin-proteosome degradation of IκB, leading to the release of NF-κB dimers, which then translocate into the nucleus to activate transcription from the HIV-1 long terminal repeat (LTR) [21,55,65,66]. Although TNF-α may be important in regulating immune response and inducing survival and regeneration in HSCs [67,68], impaired regulation in HIV-1 infection and TNF-α-induced reactivation of HIV-1 may reduce regenerative capacity, alter quiescence status, and diminish HSC population [22,69]. The adverse effects of activation of the NF-κB classical pathway include inducing secretion of inflammatory cytokines, especially via inflammasomes, induction of adhesion molecules on endothelial cells promoting leukocyte activation and transmigration, stimulating thrombotic effects, and oxidative stress [70,71,72,73], as shown in Figure 2.

Despite having limited surface levels of HIV receptors and co-receptors compared to differentiated CD4+ cells, HSCs remain susceptible to infection, highlighting the direct impact of the virus on these critical cells involved in blood cell formation [49]. This direct interference with HSC function contributes to hematological abnormalities, including chronic anemia observed during chronic HIV-1 infection [23,74,75,76]. Studies evaluating hematopoiesis in patients undergoing HIV treatment also support a direct role of the virus in inducing anemia [77,78]. Bone marrow from HIV+ patients receiving ART but without adequate response, so-called immunologic non-responders, demonstrates a decrease in the number of HSCs compared with those with response to therapy [79].

The study by Li et al. (2022) utilized humanized mice as a robust animal model to investigate the impact of chronic HIV-1 infection on HPCs [45]. The investigators found that HIV-1 infection significantly depleted the population of CD34+CD38- early HPCs in the bone marrow of humanized mice. This depletion was associated with a simultaneous expansion of intermediate CD34+CD38+ HPCs [45]. The study highlights the preferential depletion of early HPCs during chronic HIV-1 infection, shedding light on the potential mechanisms contributing to hematological alterations in PLWH.

### 3.2. Other Mechanisms of HIV Impairment of Hematopoietic Stem Cells beyond the TNF-α-Dependent NF-κB Pathway

When HIV infects HSCs, continued replication and activation of cellular death pathways leads to increased virion release, death of HSCs, and reduced production of red blood cells, resulting in anemia [80]. HSCs that are latently infected and have the ability to self-renew lead to the expansion of the latent HIV-1 reservoir, which becomes reactivated and induces cellular death when these cells differentiate [80]. A humanized mouse model study of the HIV viral protein *Nef* showed that *Nef* modulated the expression of 176 genes involved in hematopoietic cell development, including the downregulation of genes involved in the expression of CD34+ CD38− hematopoietic stem/progenitor cells and blocked human T-cell development at the progenitor level [81]. Another mechanism that may be involved in impairing HSCs is the regulation of period circadian clock 2 (Per2) on HPCs. Bordoni et al. found that HSCs of PLWH with a lower CD4 T cell count had a lower relative telomere length and reduced white progenitor colonies compared to those with a higher CD4 T cell count [82]. Compared with healthy individuals, PLWH had higher expression of Per2 on HSCs, which correlates with reduced relative telomere length. Their data suggests that Per2 is overexpressed in those with HIV infection, contributing to an impaired immune reconstitution. Sirtuin 1, an inhibitor of Per2, is downregulated in HSCs of PLWH [82]. There is still limited information from the literature reporting on underlying mechanisms that promote HSC dysfunction.

### 3.3. Effect of HIV-1 Infection on Erythropoietin Production

The dysregulation of EPO emerges as a pivotal factor in the context of HIV-associated chronic anemia. Recent investigations highlight the insufficient production of EPO and an attenuated response to its physiological action in HIV patients. Notably, the coexistence of anemia and decreased serum EPO concentration, independent of kidney damage, has been observed in many HIV patients [23]. Moreover, HIV-1 has been shown to directly impact EPO synthesis in vitro, indicating a multifaceted role of the virus in disrupting hematopoietic regulation [83].

Various mechanisms contribute to this observed reduction in EPO levels. The up-regulation of pro-inflammatory cytokines IL-1β and TNF-α by HIV is implicated in the direct downregulation of EPO expression in vitro [84]. This effect is mediated through cytokine-induced ROS, which, in turn, interferes with the binding affinities of EPO-inducing transcription factors [84]. The promoter site of the gene encoding the transcription of EPO is inhibited by GATA binding protein 2 and the NF-κB-dependent pathway [85]. As discussed earlier, HIV-1 infection of T cells and HSCs activates the NF-κB dependent pathway, resulting in impaired hematopoiesis, the production of inflammatory cytokines that further exacerbate and downregulate EPO production.

## 4. The Inflammatory Milieu in HIV Contributes to Chronic Anemia and Associated Adverse Outcomes

The chronic nature of HIV-1 infection leads to sustained immune activation and inflammatory processes, which can negatively impact the hematopoietic microenvironment. Inflammatory responses triggered by the virus may disrupt the normal functioning of hematopoietic progenitors, affecting their survival, proliferative capacity, and differentiation. Emerging evidence suggests that chronic inflammatory cytokine signaling can lead to functional exhaustion of HSCs [86,87].

The pathogenesis of chronic anemia in PLWH involves a complex interaction of chronic immune activation, iron dysregulation, and persistent inflammation, contributing to adverse outcomes. Abioye et al. (2020) reported that chronic immune activation, particularly observed in approximately 41–47% of anemia cases, is a significant contributor, with iron deficiency anemia (IDA) accounting for 20–44% of cases [17]. Pro-inflammatory cytokines, including interleukin-6 (IL-6), TNF-α, and interferon-gamma (IFN-γ), play central roles in this process [88,89]. IL-6 induces hypoferremia via increased synthesis of hepcidin in the liver, which inhibits absorption of iron in the duodenum and upper jejunum of the small intestine [90,91]. TNF-α reduces EPO synthesis and is associated with low hemoglobin synthesis in PLWH [92,93]. IL-1β associated with reduced EPO synthesis and blockade of IL-1β is associated with reduced incidence of anemia and improvement of hemoglobin levels [94].

Activated immune cells, such as macrophages and neutrophils, release these cytokines, which inhibit erythropoiesis and directly suppress red blood cell production [89]. Similarly, higher sCD14 levels and an increased count of CD14(dim)CD16(+) cells, often referred to as “patrolling” monocytes, have been observed in anemic PLWH [95]. The persistent release of pro-inflammatory cytokines creates a state of chronic inflammation, hindering the body’s ability to produce an adequate number of red blood cells. Additionally, hepcidin, the master regulator of iron homeostasis, is upregulated by these inflammatory signals [19]. Hepcidin obstructs intestinal iron absorption and causes iron retention in reticuloendothelial cells, resulting in restricted iron availability for erythropoiesis, shortened erythrocyte lifespan, and suppressed erythropoietin response to anemia [89].

Observational studies, such as those by Roldan et al. (2017) and Pereira et al. (2022), suggest that despite achieving viral suppression with the use of ART, sub-optimal inflammation persists in people with HIV who have chronic anemia [93,96] but this does not occur in individuals without chronic anemia [96]. This suggests that chronic anemia in HIV contributes to persistent inflammation beyond ART. However, the underlying mechanisms remain unclear.

**Figure 3:**
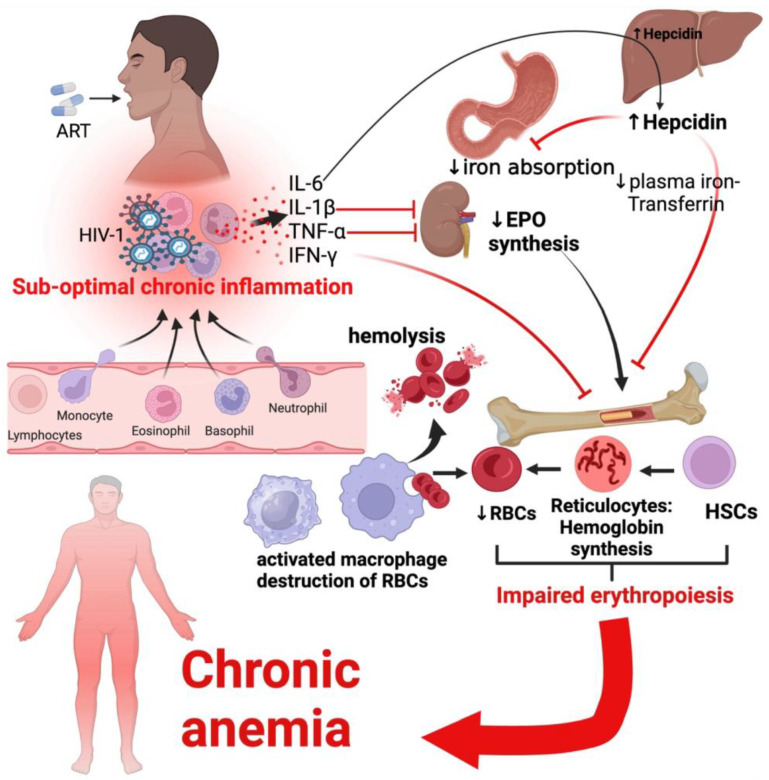
Inflammatory mechanisms of chronic anemia in treated HIV infection. Inflammatory cytokines produced by activated and HIV-1-infected cells inhibit EPO synthesis and increase hepcidin synthesis in the liver, resulting in a reduction in erythropoiesis, leading to anemia. Activated macrophages destroy RBCs. HSCs, hematopoietic stem cells; RBCs, red blood cells; EPO, erythropoietin; TNF-α, tumor necrosis factor alpha; ART, antiretroviral therapy.

Inflammation, fuelled by pro-inflammatory cytokines and immune cells, leads to chronic anemia by multiple mechanisms, as shown in Figure 3. Firstly, the inhibition of erythropoiesis directly impacts the production of red blood cells. Secondly, the shortened lifespan of erythrocytes further exacerbates the decline in red blood cell levels [19]. Additionally, the suppressed erythropoietin response to anemia hampers the body’s ability to stimulate red blood cell production in response to low hemoglobin levels [97]. Inflammation contributes to the complex pathogenesis of chronic anemia in PLWH, leading to adverse outcomes such as a more pronounced systemic inflammatory profile, increased risk of co-infections like tuberculosis, and higher mortality rates [98,99,100].

In PLWH, monitoring inflammatory markers, such as C-reactive protein (CRP), interleukin-6 (IL-6), and D-dimer, is crucial for predicting adverse outcomes, particularly in the context of chronic anemia [101,102]. Studies have established that elevated levels of CRP and IL-6 are associated with a more pronounced systemic inflammatory profile, linking inflammation to chronic anemia in PLWH [102]. The significance of these inflammatory markers extends beyond mere association, as they are linked to adverse clinical outcomes, including a heightened risk of tuberculosis (TB), immune reconstitution inflammatory syndrome (IRIS), and mortality in PLWH [98,99,103].

Consistent evidence from various studies further strengthens the link between anemia and adverse outcomes. The findings from a study conducted in Zambia revealed a significant association between a hemoglobin level lower than 8.5 g/dL persisting for six months or more and a hazard ratio of 4.5 for death [104]. This underscores the prognostic importance of anemia in the context of HIV.

Remarkably, the severity of anemia plays a crucial role in predicting AIDS-related mortality. These findings highlight the graded impact of anemia on disease outcomes in HIV patients [105]. A study conducted during the HAART era in the United States demonstrated a substantial 55% increased risk of neurocognitive disorders among individuals with anemia compared to their non-anemic counterparts [105]. This suggests a potential neurological impact of anemia in the HIV population, contributing to the spectrum of complications [105].

## 5. Chronic Anemia in HIV Is Associated with Chronic Kidney Disease and Cardiovascular Disease

### 5.1. Chronic Anemia and Chronic Kidney Disease in HIV

Several factors, including HIV-associated nephropathy, erythropoietin deficiency, chronic inflammation, and the nephrotoxic effects of antiretroviral medications, contribute to the heightened risk of chronic kidney disease (CKD) in the presence of chronic anemia [106,107]. HIV-related immunosuppression and comorbidities such as opportunistic infections further amplify the impact of anemia on renal health [108].

PLWH face an elevated risk of CKD, often associated with non-communicable diseases (NCDs) like diabetes and hypertension [109]. The prevalence of kidney disease in PLWH is three to five times higher than in HIV-negative individuals [110]. CKD and chronic anemia in this population are associated with adverse outcomes, including increased morbidity and mortality, and are linked to cardiovascular disease (CVD) [111]. Anemia is a common complication in both CKD and HIV infection, associated with an elevated risk of cardiovascular events and all-cause mortality [112]. In CKD, anemia, linked to reduced erythropoietin production, contributes to cardiovascular risk by promoting markers of endothelial activation and left ventricular hypertrophy [113].

**Figure 4:**
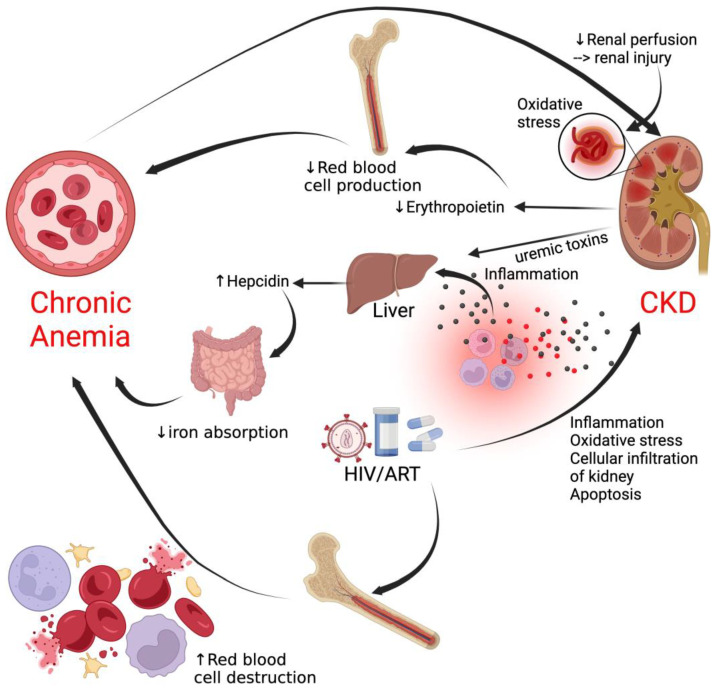
Chronic anemia and chronic kidney disease in HIV. Chronic anemia contributes to CKD development through reduced renal perfusion, leading to renal injury. However, in the context of HIV, CKD is a common complication that contributes to chronic anemia. CKD, chronic kidney disease.

Anemia exacerbates the progression of HIV-associated nephrotoxicity, leading to structural and functional changes in the kidneys and promoting the development of CKD [114]. Furthermore, erythropoietin deficiency, a common feature in HIV-related anemia, not only contributes to inadequate red blood cell production but is also implicated in renal injury, creating a dual mechanism through which anemia may impact kidney health [115], as shown in Figure 4.

Chronic inflammation and immune activation characteristic of HIV infection create a pro-inflammatory environment, directly affecting renal function and contributing to CKD. Anemia, often associated with inflammation, may further intensify these processes, exacerbating renal damage [116,117,118]. Over time, inflammation leads to scarring in the glomeruli, which can sometimes lead to chronic kidney disease (CKD) or end-stage renal disease (ESRD) [119,120].

Although chronic anemia in PLWH may contribute to the development of CKD, more often than not, anemia mostly occurs in the context of CKD due to the reduced production of erythropoietin and is worsened as CKD progresses [121]. In addition, both HIV infection and impaired kidney function synergize to increase the risk of chronic anemia [106,122].

### 5.2. Chronic Inflammation Increases the Risk for Cardiovascular Disease in HIV

Chronic inflammation contributes to persistent anemia and cardiovascular disease, particularly in the context of HIV. The elevated risk of cardiovascular events among people with HIV is primarily attributed to sustained inflammation [123,124]. Inflammatory mediators play a significant role in contributing to endothelial dysfunction, atherosclerosis, and an increased susceptibility to thrombotic events [125]. This chronic inflammatory state, combined with traditional cardiovascular risk factors, exacerbates the overall cardiovascular burden in individuals living with HIV [18,126]. Approximately 30% to 40% of patients presenting with acute myocardial infarction (AMI) have anemia either upon admission or during their hospital stay [7,127]. Chronic anemia, whether occurring independently or in conjunction with other comorbidities, is associated with adverse outcomes [7,127]. A study by Yan et al. (2023) demonstrated that inflammation correlated positively with iron deficiency anemia and increased the risk for mild left ventricular systolic dysfunction [128]. Studies consistently emphasize that the presence of dysfunctional red blood cells (RBCs) in anemic conditions significantly contributes to the exacerbation of cardiovascular disease (CVD) severity [129,130].

**Figure 5:**
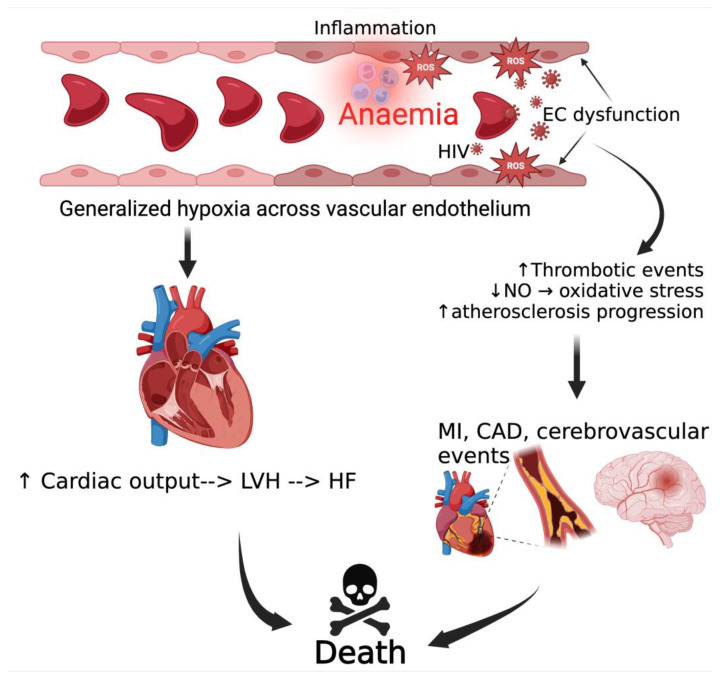
Anemia and cardiovascular disease in people with HIV. Anemia and HIV viral proteins induce endothelial dysfunction and increase cardiac output, leading to cardiovascular adverse events. LVH, left ventricular hypertrophy; HF, heart failure; MI, myocardial infarction; CAD, coronary artery disease; NO, nitric oxide; EC, endothelial cell; ROS, reactive oxygen species.

Immune-mediated endothelial dysfunction is identified as a crucial mechanism contributing to cardiovascular diseases in PLWH. HIV-related viral proteins, such as gp120, Tat, and Nef, stimulate the production of adhesion molecules and contribute to various cellular processes, including apoptosis, oxidative stress, and cytokine secretion [131,132]. Oxidative stress or increased ROS resulting from HIV viral proteins occurs through various mechanisms such as upregulation of cytochrome P450 2E1, Fenton–Weiss–Haber reaction, and activation of NADPH oxidase (NOX) enzymes type 2 and 4 (NOX2 and NOX4) [133]. Chronic anemia and immune activation in HIV patients synergistically contribute to endothelial dysfunction, creating an environment conducive to increasing the risk for cardiovascular complications [134,135,136]. HIV viral proteins also contribute to CVD development by directly inducing mitochondrial dysfunction and increasing NO production in cardiac cells, resulting in reduced myocardial contractility [137,138]. Endothelial dysfunction arising from both hypoxic effects of reduced oxygen-carrying capacity of erythrocytes and HIV viral proteins leads to increased thrombotic events, accelerating atherosclerotic processes leading to cardio-cerebrovascular adverse events and death [139,140]. Chronic anemia also increases cardiac output and increases the risk for left ventricular hypertrophy, consequently promoting heart failure [141,142,143], as shown in Figure 5.

Studies by Chennupati et al. and Wischmann et al. highlight the relationship between chronic anemia and reduced nitric oxide production due to heightened inflammation and increased formation of ROS [144,145]. The presence of chronic anemia in HIV patients adds complexity to the interaction among the immune system, endothelium, and erythropoiesis. The diminished oxygen-carrying capacity of red blood cells in anemic conditions contributes to tissue hypoxia, further aggravating endothelial impairment [139]. Janaszak-Jasiecka et al. (2021) reported that hypoxia affects nitric oxide (NO) bioavailability by influencing the control of endothelial nitric oxide synthase (eNOS) expression and activity [139]. NO is physiologically important for maintaining vascular tone and regulating inflammation and growth factors [146,147,148]. The production of NO and L-citrulline is catalyzed by the enzyme eNOS, which uses L-arginine and molecular oxygen coupled with the cofactor tetrahydrobiopterin (BH_4_) [149]. ROSs uncouple and degrade BH_4_, disrupting NO production. This disruption is caused by a decrease in the cofactor BH_4_ and a deficiency of the substrate L-Arginine, leading to the formation of superoxide instead of NO, resulting in oxidative stress [139]. Reduced NO production by endothelial cells leads to decreased vasodilation and vasorelaxation, induction of apoptosis, and necrosis, leading to endothelial dysfunction [150,151]. PLWH have been reported to have reduced levels of NO and increased inflammatory biomarkers such as IL-6 and high-sensitivity CRP with concomitant increase in the cardiovascular risk marker asymmetric dimethylarginine (ADMA) [152]. In addition to the contribution of hypoxia in disrupting NO homeostasis, HIV viral proteins reduce the expression of eNOS, resulting in increased expression of inducible NO synthase (iNOS) and more production of NO [132,153]. NO reacts with oxygen radicals, producing peroxynitrites that damage endothelial cells and contribute to the development of CVD. However, the exact mechanisms by which HIV viral proteins disrupt NO homeostasis are unclear.

Beyond the NO pathway, iron deficiency emerges as a central player in the association between chronic anemia and endothelial dysfunction. Iron deficiency, a common feature in PLWH associated with anemic conditions, contributes to the generation of ROS, fostering oxidative stress within the endothelium [154].

Recent investigations have challenged the conventional understanding linking iron deficiency anemia (IDA) with elevated endothelial NO signaling. Instead, studies, including the work of Donaway et al., 2023, suggest that endothelial alpha hemoglobin (Hbα), rather than changes in circulating hemoglobin, influences NO responses [155]. This nuanced perspective on how iron deficiency impacts vascular function may have significant implications for cardiovascular health. Moreover, additional research has revealed that specific subpopulations of iron-deficient anemic red blood cells display increased stiffness and reduced size, contributing to aberrant shear stresses and fostering vascular inflammation. Computational simulations corroborate these findings, indicating a causal relationship between the biophysical alterations of IDA red blood cells and endothelial dysfunction [129], adding depth to the understanding of cardiovascular risk in anemic HIV patients.

Recent data reaffirm the prevalence of anemia and its strong correlation with immune activation in individuals with coronary artery disease, bestowing a poorer prognosis and an increased risk of cardio-cerebrovascular death [123,130]. This association is notably amplified by chronic inflammation, traditional risk factors, and exposure to antiretroviral drugs [123,130]. The widespread impact of anemia inflammation is further recognized in patients with chronic kidney disease undergoing dialysis and those with congestive heart failure, where iron deficiency hampers cardiovascular performance [19].

## 6. Therapeutic Strategies to Control Anemia in HIV

Addressing anemia in individuals with HIV requires a comprehensive therapeutic approach due to its multifactorial origins. While ART is fundamental, its efficacy in completely resolving anemia is variable. Certain ART drugs, such as those in the integrase strand transfer inhibitor (INSTI) class or regimens containing Zidovudine, may contribute to anemia progression, necessitating careful selection of treatment regimens [42,127,156]. Opportunistic infections like tuberculosis, viral hepatitis, or bacterial infections can exacerbate inflammation and worsen anemia in HIV patients [17]. Early identification and treatment of these infections are crucial to control inflammation and support red blood cell production. Chronic diseases, particularly tuberculosis, are common contributors to anemia in this population [36,157].

Nutritional considerations play a pivotal role in managing anemia among individuals with HIV/AIDS. Adequate nutrition, including iron, Vitamin B12, and folate, is essential to counteract malnutrition-associated anemia. Nutritional counseling and support are particularly important in resource-limited settings to effectively manage anemia. Iron supplementation poses challenges in the context of inflammation; oral supplements may exacerbate inflammation, while intravenous iron therapy could be considered once inflammation is under control [158,159,160,161]. Combining iron with erythropoiesis-stimulating agents (ESAs) has shown benefits in other conditions, but its efficacy and safety in HIV-related anemia warrant further investigation through dedicated clinical trials [162,163].

A novel frontier in the management of anemia inflammation involves hepcidin-modifying agents, currently in phase III clinical trials. Modulating hepcidin levels holds promise for enhancing iron availability in HIV-related anemia, although more research is needed to translate these insights into effective interventions [164]. This novel therapeutic approach aims to antagonize hepcidin function and to mobilize iron from macrophages to deliver it for erythropoiesis.

Furthermore, exploring innovative strategies for anemia in HIV patient treatment may involve targeting the cytokine network. ω-3 poly-unsaturated fatty acids exhibit promise by downregulating TNF-α and IL-6 production, showing efficacy in inflammation-associated anemia in HIV and other inflammatory diseases like rheumatoid arthritis and diabetes mellitus [165]. Notably, specific interventions such as anti-TNF therapy for mild anemia in conditions like inflammatory bowel disease and rheumatoid arthritis have not been explored in the context of HIV-related mild anemia, representing a potential area for future investigation [6,19,89].

## 7. Future Perspectives

Despite extensive research on the hematological complications of HIV, there is a need for more in-depth exploration of specific mechanisms and pathways involved in the interaction between chronic immune activation, inflammation, and dysregulated iron metabolism, leading to persistent anemia. Additionally, limited attention has been given to understanding the impact of HIV strains on the dynamics of hematopoietic progenitor cells, specifically the CD34+. Further investigation into these areas may provide valuable insights into the pathogenesis of hematological complications in PLWH, potentially paving the way for more targeted therapeutic interventions and management strategies.

Further, there is a need for more comprehensive research elucidating the specific molecular and cellular mechanisms underlying the relationship between chronic anemia and immune-mediated endothelial dysfunction in the context of HIV. The exact pathways through which chronic anemia exacerbates inflammation and contributes to cardiovascular complications remain incompletely understood. Additionally, the literature could benefit from studies that explore the impact of different antiretroviral drug regimens on the development and progression of cardiovascular disease in HIV patients with chronic inflammation and persistent anemia. Addressing these gaps in the literature is crucial for developing targeted interventions and improving the overall cardiovascular health outcomes for individuals with HIV and concurrent chronic inflammatory and anemic conditions.

Understanding the impact of different antiretroviral drug regimens on inflammatory markers in anemic patients with suppressed viral loads is crucial. Prospective studies should investigate long-term consequences, including incident tuberculosis and mortality, to inform early interventions. Biomarkers predicting treatment success and assessing interventions beyond antiretroviral therapy are essential. Clinical trials validating biomarkers like the IL-6 and D-dimer score for predicting non-AIDS-related morbidity and mortality would advance therapeutic strategies. An integrated approach combining basic science, clinical research, and translational studies is key to advancing our understanding and management of chronic anemia and inflammation in PLWH.

## 8. Conclusions

In conclusion, the prevalence of HIV globally underscores the significance of understanding and addressing its associated hematological complications, with chronic anemia being a common and impactful manifestation. This comprehensive review has illuminated the complex interaction between chronic anemia, inflammation, and cardiovascular disease in individuals living with HIV. The multifactorial mechanisms driving chronic anemia in HIV, including hematopoietic stem cell impairment, dysregulated erythropoietin production, and persistent immune activation, underpins the complexity of this interaction. Chronic inflammation emerges as a central link linking persistent anemia and cardiovascular complications, adversely affecting erythropoiesis, erythrocyte lifespan, and the cardiovascular system. The heightened risk of co-infections, tuberculosis, and mortality further emphasizes the clinical significance of chronic anemia in individuals with HIV. Additionally, the review highlights the aggravating impact of chronic anemia on HIV-associated nephrotoxicity and its contribution to cardiovascular risk. Recognizing the cardinal role of chronic inflammation in these interrelated pathways emphasizes the need for universal understanding and targeted interventions to address the evolving healthcare needs of individuals living with HIV. This review provides valuable insights for clinicians and researchers, paving the way for enhanced clinical management approaches and targeted interventions to mitigate the burden of chronic anemia and its cardiovascular implications in the context of HIV.

## Data Availability

All data used in the writing of this manuscript are contained within. No original data have been used.
